# Gamma Knife Radiosurgery to Four Brainstem Lesions After Whole Brain Radiation Therapy

**DOI:** 10.7759/cureus.17226

**Published:** 2021-08-16

**Authors:** Mark E Bernard, William St Clair, Damodar Pokhrel

**Affiliations:** 1 Radiation Oncology, University of Kentucky, Lexington, USA; 2 Physics, University of Kentucky, Lexington, USA

**Keywords:** brainstem lesion, stereotactic radiosurgery srs, single fraction stereotactic radiosurgery

## Abstract

Our patient was a 58-year-old female with a history of extensive stage small cell lung cancer initially diagnosed in November 2018. She received palliative radiation to the right hip and whole brain in December of 2018 and then received chemotherapy. Unfortunately, in October 2019, the repeat brain magnetic resonance imaging (MRI) showed recurrent lesions and she was referred for Gamma Knife Radiosurgery (GKRS). At the time of the treatment, she was found to have four brainstem lesions as well as a left frontal lobe and a right frontal lobe lesion. She completed GKRS to all six lesions without any neurological complications seen in her short-term follow-up. This case report adds to the growing body of literature showing safety of GKRS for multiple brainstem lesions.

## Introduction

Brainstem metastasis accounts for approximately 5% of intracranial metastasis [1]. They are associated with low survival rates and are considered a poor prognostic sign [2]. The location of these metastases is challenging because if left unmanaged, they can lead to rapid neurological decompensation that can lead to death [2]. It is considered a high-risk area for treatment-related morbidity.

Treatment options for brainstem metastasis can be challenging given the risk of neurological morbidity [1-2]. Surgical resection is typically avoided due to high risk of complications, leaving radiation as an efficacious treatment option. Whole brain radiation therapy (WBRT) is a safe treatment option, however, progressive or recurrent lesions in the brainstem can occur, which further narrows treatment options.

Stereotactic radiosurgery (SRS) has been shown to be a safe and efficacious treatment for brain metastasis [3]. This treatment can be safely given whether the patient has had prior whole brain radiation therapy or not [3]. However, Quantitative Analyses of Normal Tissue Effects in the Clinic (QUANTEC) has limited the brainstem constraint for single-fraction treatment to 12.5 Gy [1]. This dose limit may result in an inadequate dose to ablate the gross metastatic disease in or near the brainstem.

This case report shows the brainstem tolerance may be much higher after our patient received Gamma Knife Radiosurgery (GKRS) to four brainstem lesions, in addition to two frontal lobe lesions.

## Case presentation

Our patient initially presented at the University of Kentucky Radiation Oncology Department in November 2018 after being diagnosed with extensive small cell lung cancer at an outside hospital. She had brain metastasis with one nearly compressing the fourth ventricle and another in the pituitary gland. She completed a course of whole brain radiation and palliative radiation to the right hip consisting of 30 Gy in 10 fractions to each site and then went on to receive six cycles of cytotoxic chemotherapy. She represented to us in October 2019 with a repeat brain MRI showing four sub-centimeter lesions in the brainstem, but it was decided to observe these lesions given the size. However, she had another magnetic resonance imaging (MRI) in December 2019 that showed progression of multiple brain lesions such as a 10 mm × 9 mm lesion in the posterior left frontal lobe, and a 3 mm × 3 mm enhancing lesion in the right lateral pons. It was then decided for her to receive Gamma Knife Radiosurgery (GKRS).

**Figure 1:**
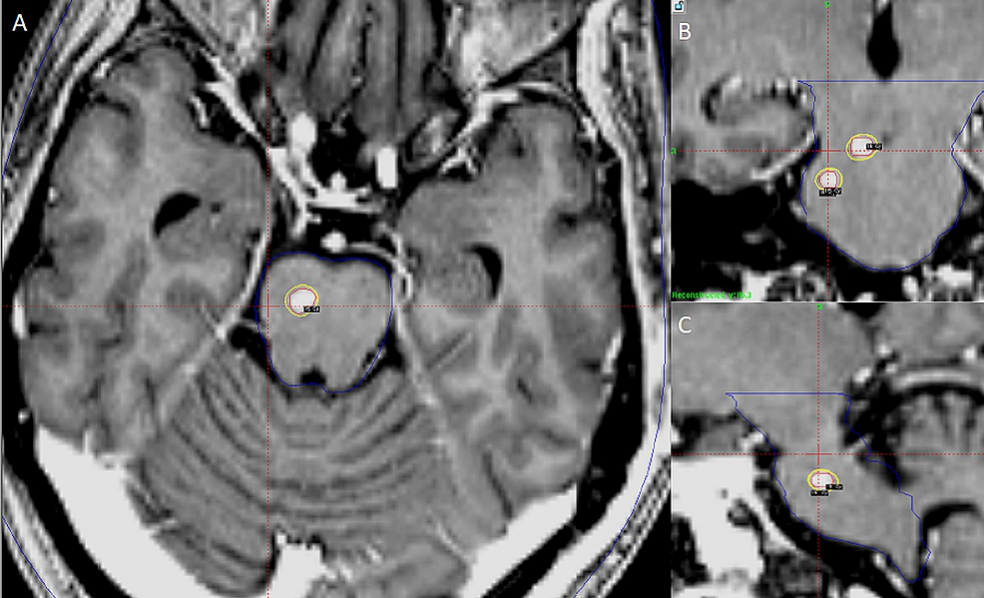
Axial and coronal views of a high-resolution MPRAGE image of the brain used for Gamma Knife Radiosurgery (GKRS) planning in the treatment of superior brainstem lesions (solid green matrix), brainstem contour (blue) and the 15 Gy prescription isodose line (yellow) to each lesion is shown. A: Axial images within the pons B: Sagittal images within the brainstem C: Sagittal images within the brainstem

On the day of treatment, our patient presented to the GKRS center at the University of Kentucky where she was fitted with a Leksell stereotactic head frame. After placement of the head frame, the patient was taken to the Department of Radiology for a high-resolution 1 mm cuts MPRAGE MRI scan. When the MRI scan was completed, the patient returned to the GKRS center and the information acquired from the MRI was subsequently downloaded to the GammaPlan software (GammaPlan Version 11.1, Elekta Inc., Stockholm, Sweden). This information was utilized to identify the patient's new brain metastases and was utilized to generate a GKRS plan for dosimetry and treatment. A total of six lesions were identified and treated with a summary of them in Table 1. Four of the six lesions were located in the brainstem (Figure 1). Following the conclusion of the GKRS procedure, the head frame was removed.

**Table 1:** Location, size, and dosimetry information of the treated lesions

Lesion	Location	Lesion size (mm)	Dose (Gy)	Isodose Line	Conformity Index
1	Left Frontal Lobe	7.3 × 10.0 × 9.0	24	50%	1.85
2	Superior Brainstem	6.0 × 6.0 × 4.3	15	70%	1.35
3	Superior Brainstem	3.0 × 3.5 × 3.0	15	70%	1.44
4	Inferior Brainstem	8.5 × 8.0 × 7.5	15	65%	1.42
5	Inferior Brainstem	2.5 × 2.0 × 3.0	15	65%	1.51
6	Right Frontal lobe	2.5 × 3.0 × 2.5	24	85%	1.95

The maximum point dose to the brainstem was 17.95 Gy (<18.0 Gy). The volume receiving 12.5 Gy of brainstem was 1.1 cc. The D05%, D25%, and D50% of the brainstem were 11.5 Gy, 4.5 Gy and 2.7 Gy, respectively. Mean brainstem dose was 3.6 Gy. The D95% of the brainstem was less than 0.5 Gy. After treatment was completed, she received 12 mg of dexamethasone.

She was seen approximately five weeks later and no changes in her mental status were reported. She did have restaging computed tomography (CT) scans that showed progression of her thoracic disease with superior vena cava syndrome. She was placed on nivolumab and ipilimumab and started on palliative radiation therapy. Unfortunately, she was admitted to the inpatient services, her thoracic malignancy progressed, her airway closed, and she expired, which was two months from completing her GKRS treatment. During this time, while she had nausea requiring antiemetic medications, she did not develop focal deficits or neurological side effects thought to be attributable to her GKRS treatment. However, during her hospital stay, she was placed on steroid treatment.

## Discussion

Management for multiple brain metastasis can consist of surgical resection, WBRT, or SRS treatments [1-2]. Previous trials have shown either a survival benefit, distant brain control benefit, or local control benefit using these techniques [3-5]. However, management for brainstem metastasis can be challenging due to the concern for side effects. Surgical resection for brainstem metastasis is not common due to the risk of perioperative morbidity. WBRT is a safe option for these patients; however, these lesions can progress causing addition therapy to be warranted. GKRS is a great option because it can deliver an ablative dose in a single fraction, however, there can be concern regarding the safety of this technique, especially after WBRT. A further complexity is management for multiple brainstem metastasis after WBRT as presented in this case report.

Several retrospective reports have shown the efficacy of GKRS for brainstem metastasis. However, the majority of these patients have had one or two lesions. The University of Virginia (UVA) reported on 61 patients who received GKRS for brainstem metastasis or arteriovenous malformations [1]. The median prescription dose was 18 Gy (range: 13-25 Gy). Approximately 16% of patients experienced clinical side effects and 28% of patients experienced complications. Albeit, the majority of these patients had either one (85%) or two (13%) lesions. Only one patient had three lesions in the brainstem.

The University of Pittsburgh published a series of 27 lesions in 26 patients who received GKRS to the brainstem. The median dose was 16 Gy (range: 12-20 Gy) [2]. The majority of the patients had a single lesion in the brainstem. While the local control rate was 95%, four patients experienced transient dizziness, nausea, vomiting which resolved within hours, and three patients experienced a seizure which was controlled with anti-epileptic medications.

Kawabe et al. also reported on 200 patients treated with brainstem lesions of which 15 (7.5%) had two or more lesions [6]. One patient had four and one patient had five lesions in the brainstem. The median dose was 18 Gy (range: 12-25 Gy). Only one patient experienced a severe GKRS complication and six patients needed oral steroids for one to three months due to MRI showing an increase in peritumoral edema.

Our case adds to the limited number of patients treated with GKRS for multiple brainstem metastasis, after WBRT. Despite the prior dose to the brainstem, our patient did not have any complications related to treatment. The maximum dose of the brain was above the QUANTEC recommended guidelines (<12.5 Gy). While ours was 17.95 Gy, only 1.1 cc of the brainstem received 12.5 Gy. UVA recently showed that dose to the brainstem (D05%, D95%, and median) was predictive of increased complications over time [1]. In their retrospective cohort, the median D05% of those who did not experience side effects was 6.3 Gy (range: 0.29-12.7 Gy) and the median D95% was 0.24 Gy (range: 0.06-2.07 Gy). For this patient, the D05% was 11.7 Gy and D95% was less than 0.5 Gy. In short, while our patient did not experience any acute side effects due to dose to the brainstem, it may be associated with the relatively small lesion size and thus small doses to the brainstem. It’s important to note, our patient had prior WBRT.

This case report has weaknesses. The patient’s primary was not controlled, and she eventually succumbed to her disease within two months of her GKRS treatment. Therefore, long-term side effects could not have been evaluated. Also, there was no post-treatment MRI done and thus radiographic complications were not evaluated. However, even if there were radiographic complications, they did not manifest clinically. Regardless, given the short survival of the patient, whether or not to treat brainstem metastasis should be made with respect to the expected survival of the patient.

## Conclusions

GKRS for brainstem lesions is an efficacious treatment option and maybe a feasible option for patients with multiple brainstem metastasis after WBRT. Further data is needed to establish more concrete dose constraints for the brainstem in this setting. While evaluating patients for this option, close attention should be paid to the size of the lesions and the dose going to five percent and ninety-five percent of the brainstem. Future prospective clinical trials can consider recording dosimetric metrics to the brainstem to help establish modern dose constraints.
